# MicroRNA-708-5p acts as a therapeutic agent against metastatic lung cancer

**DOI:** 10.18632/oncotarget.6594

**Published:** 2015-12-14

**Authors:** Xiaoping Wu, Tianchi Liu, Ou Fang, Wenhua Dong, Fengjun Zhang, Lindsey Leach, Xiaohua Hu, Zewei Luo

**Affiliations:** ^1^ Laboratory of Population and Quantitative Genetics, Institute of Biostatistics, State Key Laboratory of Genetic Engineering, School of Life Sciences, Fudan University, Shanghai, China; ^2^ School of Biosciences, University of Birmingham, Birmingham, UK

**Keywords:** non-small cell lung cancer, therapeutic agent, MicroRNA-708-5p, metastasis, p21

## Abstract

MicroRNAs (miRNAs) have recently been recognized as targets for anti-metastatic therapy against cancer malignancy. Development of effective miRNA mediated therapies remains a challenge to both basic research and clinical practice. Here we presented the evidence for a miR-708-5p mediated replacement therapy against metastatic lung cancer. Expression of miR-708-5p was substantially reduced in metastatic lung cancer samples and cancer cell lines when compared to non-metastatic counterparts. Expression of the miRNA suppressed cell survival and metastasis *in vitro* through its direct target p21, and inhibited the PI3K/AKT pathway and stem cell-like characteristics of lung cancer cells. Systemic administration of this miRNA in a mouse model of NSCLC using polyethylenimine (PEI)-mediated delivery of unmodified miRNA mimics induced tumor specific apoptosis. It also effectively protected the tested animals from developing metastatic malignancy without causing any observed toxicity. The findings strongly support miR-708-5p as a novel and effective therapeutic agent against metastatic malignancy of non-small cell lung cancer.

## INTRODUCTION

Lung cancer is a major cause of death among malignant diseases, primarily due to its high incidence, malignant behavior and lack of effective treatments [1]. Non-small cell lung cancer (NSCLC), accounting for ∼85% of lung cancer incidence, is often diagnosed at an advanced stage and has a poor prognosis [2]. Although surgery, radiation therapy and chemotherapy can control many other primary tumors effectively, these treatments have been ineffective in curbing the metastatic spread of lung cancer cells [3], creating an urgent need for development of more effective therapies against the highly metastatic disease.

miRNAs are small non-coding RNAs responsible for regulating more than 70% of human genes. They have been increasingly identified as novel biomarkers or therapeutic targets [4–7]. miRNAs may act as either oncogenes or tumor suppressors, depending on the type of tumor or the cellular context [8, 9]. Inactivation of oncogenic miRNAs [10, 11] or restoration of tumor suppressing miRNAs [12–14] has a great potential for cancer treatment, and both strategies have been tested in clinical trials [15–19]. However, there has been little or no research reported in the literature on miRNA mediated prophylactic therapies against lung cancer metastasis.

We detected several significantly differentially expressed miRNAs in NSCLC cell lines from TaqMan Human MicroRNA Array data [20]. Among them, miR-708-5p stood out as a highly expressed miRNA in non-metastatic lung cancer cell lines when compared with highly metastatic cells. We thoroughly exploited this miRNA for its involvement in tumor formation, metastasis and apoptosis of NSCLC cells, and tested its potential as a therapeutic agent against metastatic NSCLC.

## RESULTS

### miR-708-5p expression is associated with metastatic status of NSCLC cell lines and tissue samples

**Figure 1:**
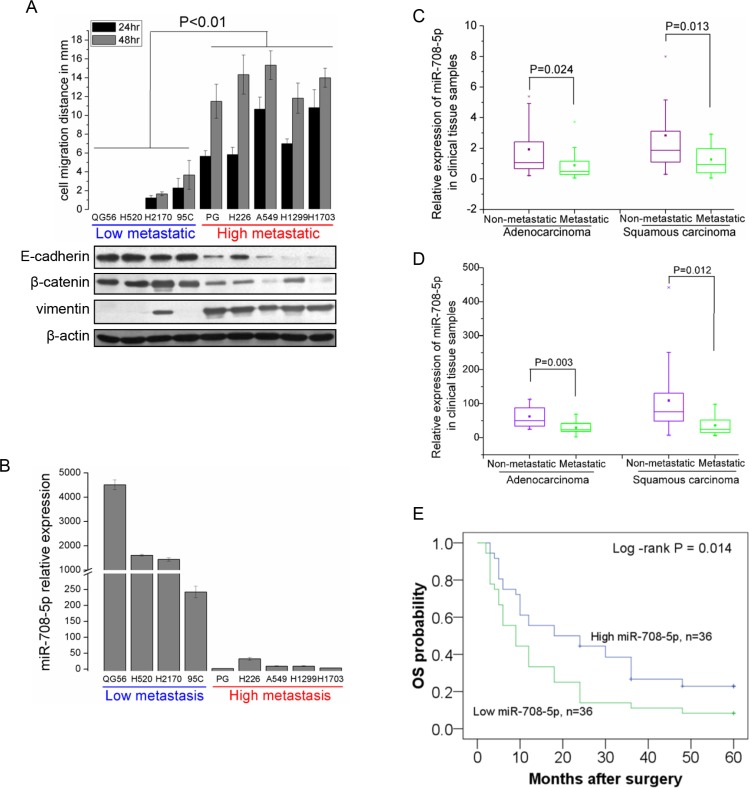
miR-708-5p levels correlate inversely with metastatic ability of NSCLC cell lines and NSCLC tissue samples (**A**) Assay of metastatic ability in nine NSCLC cell lines. Upper: migration distance (mm) of nine NSCLC cell lines (QG56, H520, H2170, 95C, PG, H226, A549, H1299 and H1703). Cells were placed in six-well cell culture plates and their migrations were observed at 24 and 48 h of the wound healing assay. Shown below is immunoblotting analysis of three representative endogenous EMT marker genes (E-cadherin, β-catenin and vimentin) in the NSCLC cell lines. In the analysis, β-actin served as an internal control. (**B**) miRNA qRT-PCR analysis showing significant suppression of miR-708-5p in high-metastatic NSCLC cell lines when compared with low-metastatic group. (**C**) Relative expression of miR-708-5p in 72 NSCLC tissue samples (36 Adc and 36 Sqc) with metastasis (*n* = 20 Adc + 20 Sqc) or non-metastasis (*n* = 16 Adc +16 Sqc). qRT-PCR analysis shows that miR-708-5p are down-regulated in metastatic tumor samples when compared with that in the non-metastatic tissue samples. (**D**) Relative expression of miR-708-5p in another 66 NSCLC tissue samples (28 Adc and 38 Sqc) with metastasis (*n* = 18 Adc + 9 Sqc) or non-metastasis (*n* = 10 Adc + 29 Sqc). All miRNA expression levels were normalized to the small nuclear RNA U6 and the Mann-Whitney-Wilcoxon test was conducted to infer statistical significance of the miRNA expression from the metastatic and non-metastatic groups. (**E**) Cohort was dichotomized based on miR708-5p median expression and presented as a five-year overall Kaplan-Meier survival curves in a panel of patients of NSCLC. Statistical treatment of the data is log-rank. *P* values are indicated on the graph.

We identified miR-708-5p for its potential role in lung tumor formation, metastasis and apoptosis, based on analysis of TaqMan Human MicroRNA Array data from NSCLC cell lines in our previous profiling of NSCLC associated miRNAs [20]. To further confirm its candidacy, we re-examined expression of miR-708-5p in nine NSCLC cell lines varying in their metastatic status. Their metastatic status was primarily defined through a wound-healing assay and in term of expression profiling of the three representative epithelial-to-mesenchymal transition (EMT) related marker genes (E-cadherin, β-catenin and vimentin), as the EMT has been established as a critical event in the invasion, progression and metastasis of epithelial cancers [21, 22]. Based on the wound-healing assay, four cells (QG56, H520, H2170 and 95C) were assigned into a low metastatic group showing low migration ability, while the other five cell lines (PG, H226, A549, H1299 and H1703) formed a high metastatic group with high migration ability (Figure 1A). These were further corroborated by the western blot assay of EMT markers (Figure 1A). qRT-PCR analysis showed that expression of miR-708-5p was markedly reduced in the high metastatic group when compared to the low metastatic ones (Figure 1B). Additionally, we analyzed miR-708-5p expression in 72 micro-dissected human NSCLC tissues (36 adenocarcinomas (Adc) and 36 squamous cell carcinomas (Sqc)) by qRT-PCR, which were divided into non-metastatic (16 Adc + 16 Sqc) and metastatic (20 Adc + 20 Sqc) groups according to the same criterion above. Basic clinical characteristics of these samples were summarized in Supplementary Table S1. Consistent with the cell line analysis, both Adc and Sqc metastasis positive tumors showed significant down-regulation of miR-708-5p when compared to the non-metastatic tumors of the two subtypes (Figure 1C, Supplementary Table S1). However, no significant difference was observed in the miR-708-5p expression level between age or gender groups (Supplementary Table S1). Profiling miR-708-5p expression of additional 66 NSCLC samples also independently confirmed the inversing correlation between miR-708-5p expression and tumor malignancy (Figure 1D). All these results strongly support the potential tumor suppressor role of miR-708-5p in lung carcinoma.

To further determine miR-708-5p expression as a prognostic biomarker, we investigated the trend of miR-708-5p expression over the overall survival (OS) among the 72 NSCLC patients (Supplementary Table S1). It can be seen from Figure 1E that OS of the patients expressing a low level of miR-708-5p had a OS median of 15.4 months, significantly lower than those expressing a high level of the miRNA (OS median of 30.0 months) (log-rank *P* = 0.014).

### Expression of miR-708-5p suppresses lung cancer invasion and metastasis *in vitro* and *in vivo*

**Figure 2:**
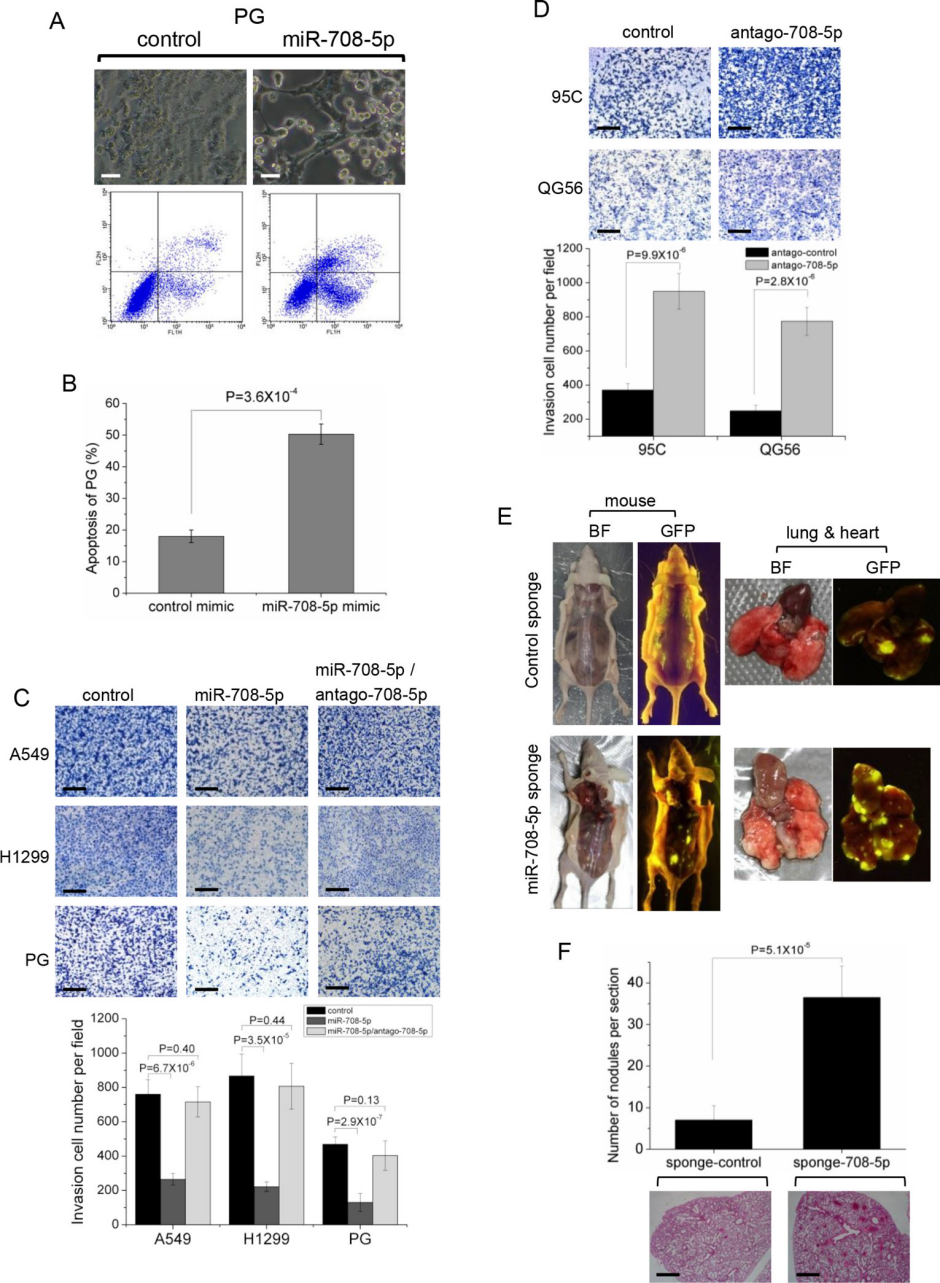
miR-708-5p induced cell apoptosis and suppressed invasive growth of lung cancer cells (**A**) Apoptosis assay was performed in the PG cells transfected with either control mimics or miR-708-5p mimics after serum deprivation for 48 h. Upper group: altered morphology of the PG cells transfected with the control or miR-708-5p mimics. The assays were assessed by phase-contrast microscopy. Scale bars, 80 μm. Lower group: apoptosis assays of the PG cells using the Annexin V/PI kit after the control or miR-708-5p mimics treatments and the assays were detected by flow cytometry. (**B**) Proportions of average apoptosis (including both early and late apoptosis) in the two comparing groups of PG cell line. (**C**) Transwell matrigel invasion assay of the A549, H1299 and PG cells 48 hours after transfected with control mimics, miR-708-5p mimics, or miR-708-5p/antago-708-5p (antagomir-708-5p). (**D**) Transwell matrigel invasion assay of the 95C and QG56 cells transfected with antagomir control or antago-708-5p. (C, D) Upper: imagines of the cells penetrating through the matrigel after stained with 0.25% Trypan Blue. Scale bars, 250 μm. Lower: means and standard deviations (s.d.) of the number of the cells that penetrated through the transwell membrane. (**E**) Inhibited miR-708-5p promotes metastasis *in vivo*. Bright-field imagines (BF) and GFP views of mice surgically examined and their lungs and hearts isolated from the mice that received caudal vein injection of the QG56 cells infected with the miR-708-5p sponge or control sponge. (**F**) The number of metastatic nodules in the lungs of mice that received caudal vein injection of the QG56 cells infected with the miR-708-5p sponge or control sponge. Upper: means and s.d. of counts of metastatic nidi in the lung tissue slices of the tested mice (*n* = 6 for each group). Lower: representative H & E stained images of lungs showing metastatic nodules. Scale bars, 500 μm.

Transfection of miR-708-5p mimics in high metastatic PG, A549 and H1299 cells induced cell apoptosis (Figure 2A, 2B and Supplementary Figure S1A, S1B). Expression of the apoptotic markers, active caspase-3 and cleaved PARP, were also found to be up-regulated in these cells after transfection with miR-708-5p (Supplementary Figure S1C). Furthermore, over-expression of miR-708-5p in these cells also resulted in weakened migration (Supplementary Figure S2A) and invasion by 3–6 fold when compared to control cells (Figure 2C), whereas down-regulation of miR-708-5p using an antagomir in QG56 and 95C cells led to a significant increase in cell migration (Supplementary Figure S2B) and invasion by 2–3 fold when compared to control cells (Figure 2D). The miR-708-5p mediated regulation of invasive growth and metastasis of lung cancer cells was further supported by the observation that the miR-708-5p-induced reduction in metastasis was completely abolished in the presence of antagomir-708-5p (Figure 2C).

The observed anti-metastasis *in vitro* motivated us to investigate miR-708-5p expression and its effects on metastasis *in vivo*. We used a lentiviral miRNA sponge to knock down miR-708-5p stably in QG56 cells with highly expressed miR-708-5p, and intravenously injected mice with the sponge-control or sponge-miR-708-5p cells. Mice in the sponge-miR-708-5p group exhibited considerably higher prevalence of metastasis than those in the sponge-control group. Tumours were found in the lung, heart and bone in the sponge-miR-708-5p group (Figure 2E). The lungs had an average of 30 visible metastases in the sponge-miR-708-5p group, whereas visible metastasis was reduced by about 70% in the sponge-control group (Figure 2F). These data strongly support miR-708-5p as a metastasis suppressor in lung cancer cells *in vivo*.

### p21 is a direct target of miR-708-5p

**Figure 3:**
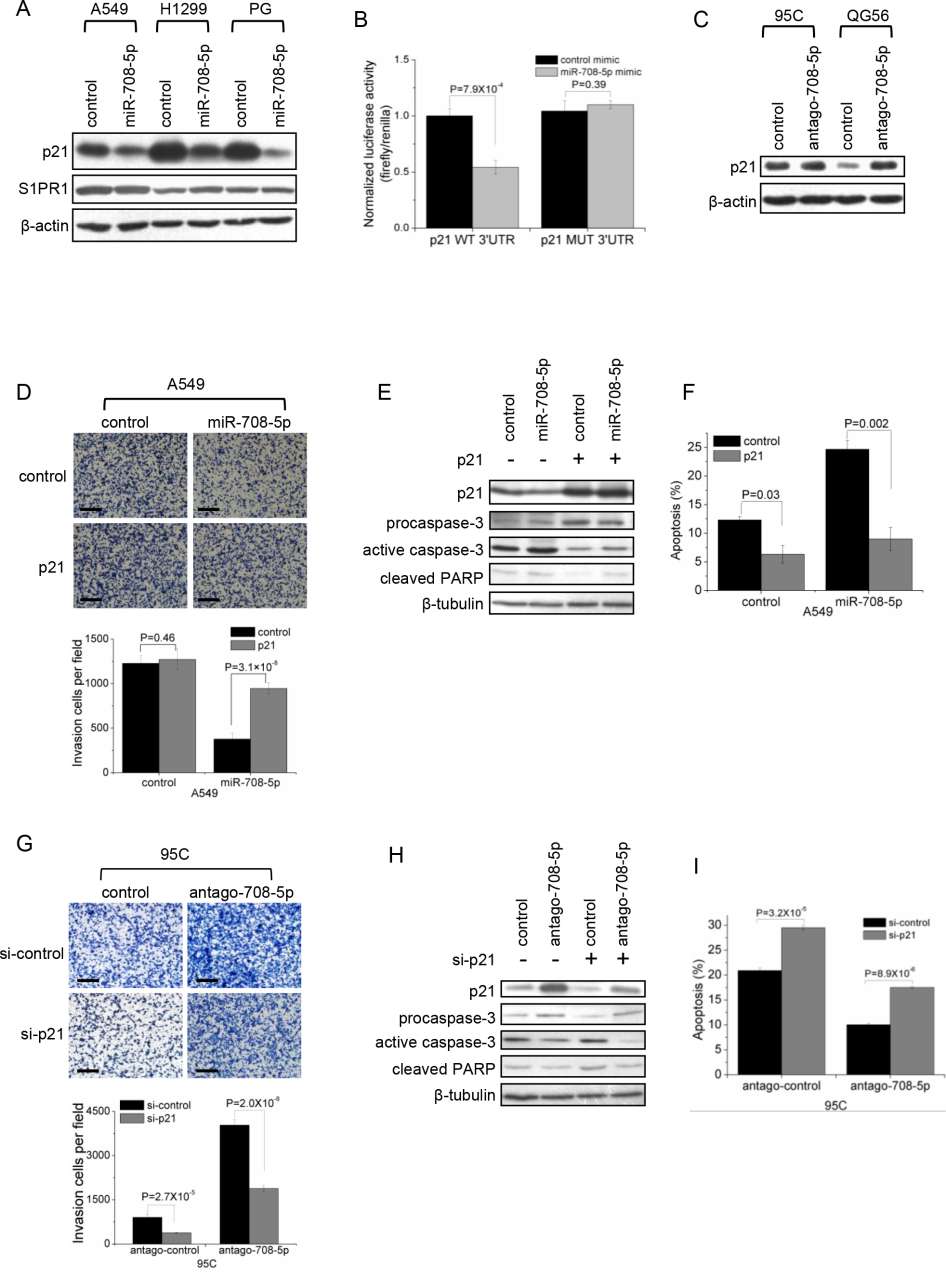
p21 is a direct target of miR-708-5p (**A**) Immunoblotting for endogenous p21 and S1PR1 proteins in the A549, H1299 and PG cells transfected with the miR-708-5p or control mimics. β-actin was used as a loading control. (**B**) Luciferase activity of the wild-type or mutant p21 3′UTR reporter genes in the 293T cells transfected with the miR-708-5p or control mimics. (**C**) Immunoblotting of p21 in the 95C and QG56 cells transfected with antago-708-5p or control. β-actin was used as a loading control. (**D**– **F**) Matrigel invasion and apoptosis assay in A549 cells co-transfected with p21 expressing vector or control vector and miR-708-5p or control mimics. (**G**– **I**) Matrigel invasion and apoptosis in the 95C cells co-transfected with antago-708-5p or antago control and p21 siRNA (si-p21) or control siRNA (si-control). (D, G) Upper: inversion imagines of the cells penetrating through the matrigel after stained with 0.25% Trypan Blue. Scale bars, 250 μm. Lower: means and s.d. of the number of the cells penetrating through the transwell membrane. (E, H) Immunoblotting of active caspase-3 and cleaved PARP in A549 (E) and 95C (H). (F, I) means and s.d. of fractions of apoptotic cells (early and late apoptosis) detected using Annexin V/PI kit in A549 (F) and 95C (I) cells.

We implemented bioinformatics algorithms, including TargetScan, miRanda and Rna22, to predict candidate targets of miR-708-5p, and detected 10 candidates: CCND3, p21, S1PR1, DRAM1, BAG1, GAS1, FLT1, MEN1, p15 and IKBKB, which were directly or indirectly associated with metastatic functions such as cell proliferation, apoptosis, cell cycle, migration, adhesion, invasion and/or cell differentiation. The dual-luciferase UTR reporter assay revealed that p21 and S1PR1 showed a highly significantly depressed 3′UTR (Supplementary Figure S3A). Only the p21 protein levels were significantly decreased in A549, H1299 and PG cells expressing miR-708-5p (Figure 3A). Further luciferase reporter assays showed that miR-708-5p significantly depressed the relative luciferase activity of the wild-type 3′UTR of p21 when compared to that of mutant 3′UTR (Figure 3B and Supplementary Figure S3B). Additionally, inhibition of miR-708-5p significantly increased the expression of p21 protein in 95C and QG56 cells (Figure 3C). These findings indicate that miR-708-5p directly regulates p21 expression through directly targeting its 3′UTR.

To test whether p21 was the direct functional mediator of miR-708-5p-induced metastasis and apoptosis of lung cancer cells, we employed an expression construct in A549 cells which encodes p21 coding sequence but lacks the 3′UTR, yielding an mRNA resistant to miRNA-mediated suppression. Ectopic expression of p21 increased migration and invasion in miR-708-5p overexpressing A549 cells but not in control cells (Figure 3D). And, reduction of active caspase-3/cleaved PARP and apoptosis cells confirmed that overexpression of p21 depressed apoptosis in both miR-708-5p expressing cells and control cells (Figure 3E, 3F, Supplementary Figure S3C). In parallel, we synthesized a short interfering RNA (siRNA) for p21 and introduced the siRNA into 95C cell line that was transfected with antagomir-708-5p. The knockdown p21 partially abolished the enhancing effects of miR-708-5p antagomir on migration and invasion of the lung cancer cells (Figure 3G). And, inhibition of p21 restored cell apoptosis in the antagomir-708-5p 95C cells by 80% compared with the corresponding control cells (Figure 3H, 3I, Supplementary Figure S3D). The bidirectional assays for engineering p21 expression both support that the ability of miR-708-5p to inhibit metastasis and promote apoptosis is attributable to its capacity to depress its target gene p21. We conclude that p21 is indeed a direct functional mediator of miR-708-5p.

### miR-708-5p induces apoptosis and suppresses cell migration by inhibiting cytoplasmic localization of p21

**Figure 4:**
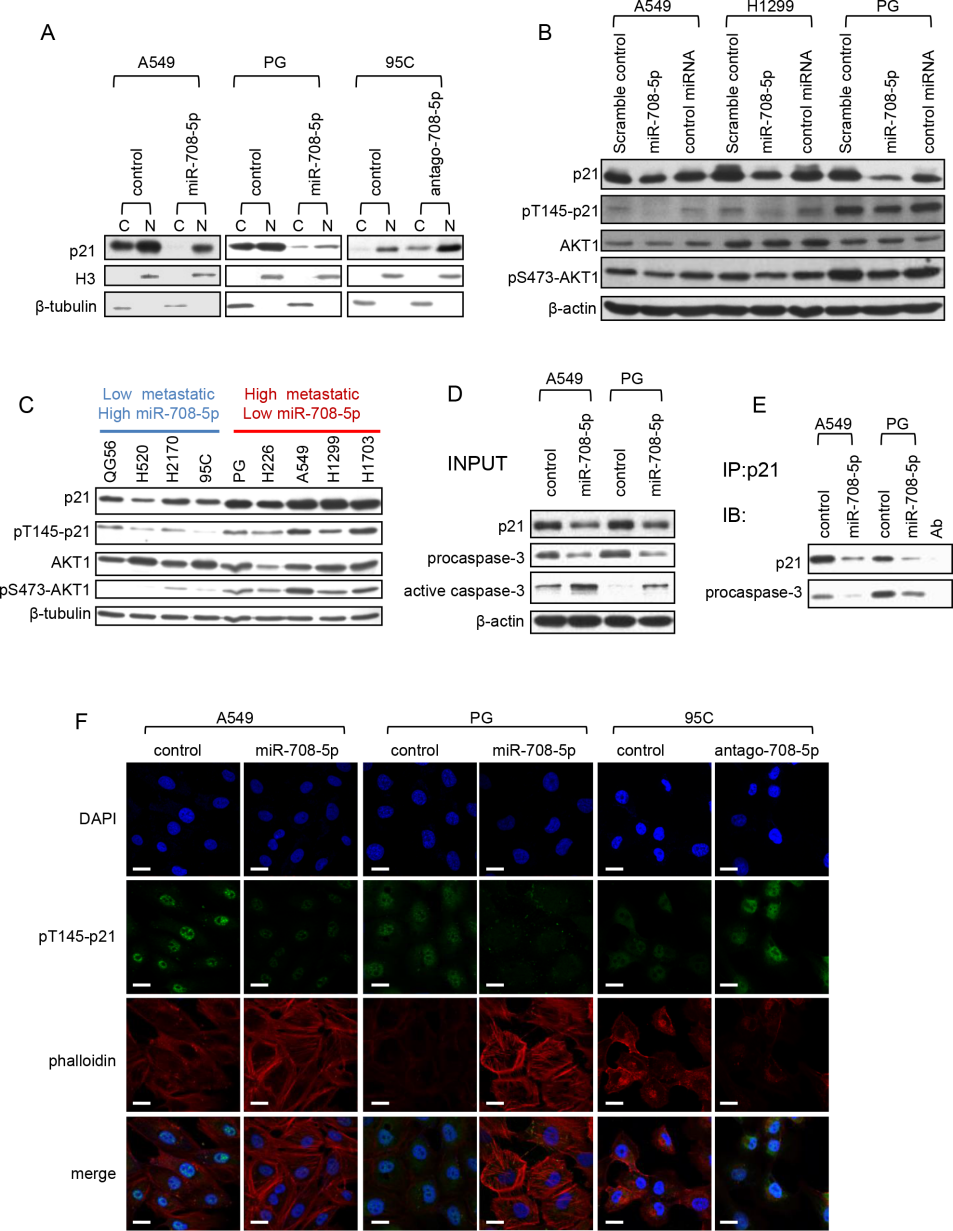
miR-708-5p inhibits cytoplasmic localization of p21 (**A**) Subcellular fractionation assay was carried out to determine the cellular localization of p21 in the A549 and PG cells transfected with miR-708-5p and control mimics, and 95C cells transfected with antago-708-5p and antago-control. Nuclear and cytoplasmic fractions were respectively subjected to immunoblotting analysis with antibody against p21. Histone 3 (H3) and β-tubulin were used as loading controls for nuclear and cytoplasmic proteins respectively. (**B**) Immunoblotting of p21, pT145-p21, AKT1 and pS473-AKT1 in the A549, H1299 and PG cells transfected with the miR-708-5p mimics, a control miRNA mimics (miR-708-3p) or scramble control mimics. β-actin was used as a loading control. (**C**) Immunoblotting of p21, pT145-p21, AKT1 and pS473-AKT1 in QG56, H520, H2170, 95C, PG, H226, A549, H1299 and H1703 cell lines. (**D**) Co-immunoprecipitation of endogenous p21 and detection of endogenous procaspase-3. The A549 and PG cells, which were serum deprived for 48 h, were stimulated by exposure to 10% serum for 6 h. A portion (5%) of the whole cell lysates was used as input for the immunoblotting analysis, and β-actin was used as a loading control. (**E**) Endogenous p21 was immunoprecipitated from 2 mg of the protein of A549 and PG cell with the p21 antibody, and probed with p21 and procaspase-3 antibodies in the western blotting assay. (**F**) Confocal images of the A549 and PG cells transfected with the miR-708-5p or control mimics for localization of pT145-p21 and cytoskeleton, and 95C cells transfected with antago-708-5p or control. The cells were synchronized after serum deprivation for 48 h and then re-stimulated with 10% serum for 6 h. Scale bars, 30 μm.

p21 can localize in the cytoplasm, where it protects cells against apoptosis [23] and inhibits Rho-kinase activity, affecting the formation of actin stress fibers [24]. Notably, cytoskeleton reorganization has been established to be essential for cell migration [25]. We firstly examined the distribution of p21 in the nucleus and cytoplasm by immunoblotting. In the A549 and PG cells transfected with miR-708-5p mimics, only was a small amount of p21 observed in the cytoplasm after serum deprivation when compared to the corresponding control cells (Figure 4A). On the other hand, cytoplasmic p21 expression was boosted in low metastatic 95C cell line when transfected with antagomir-708-5p (Figure 4A). These support that the cytoplasmic localization of p21 was impaired by miR-708-5p.

The cytoplasmic localization of p21 is additionally regulated by its phosphorylation at the Thr145 residue directed by phosphorylated AKT (pAKT, the activated form) [26]. We monitored the protein levels of pT145-p21 and pS473-AKT1 in A549, H1299 and PG cells expressing miR-708-5p. As expected, an increase in miR-708-5p expression significantly depressed the protein levels of p21, pT145-p21 and pS473-AKT1 (Figure 4B). Since pS473-AKT1 is the major effector of the PI3K/AKT pathway in cancers [27] and its protein expression was reduced in the presence of miR-708-5p, this suggests that miR-708-5p could also inhibit the PI3K/AKT pathway.

Additionally, to further confirm correlation between expression of p21, pT145-p21 and PI3K/AKT and miR-708-5p expression and metastatic ability, we used immunoblotting to evaluate the expression level of p21, pT145-p21 and pS473-AKT1 in the nine NSCLC cell lines whose expression of miR-708-5p and metastatic ability had been confirmed. The results showed that expression of p21, pT145-p21 and pS473-AKT1 was negatively correlated with expression of miR-708-5p, but positively correlated with metastatic ability (Figure 4C). We thus conclude that down-regulation of p21 was involved in miR-708-5p-suppressed metastasis.

The binding of p21 to procaspase-3 in the cytoplasm, rather than to the active caspase-3, is essential for resistance to Fas-mediated apoptosis [28]. To evaluate whether the blocked p21 cytoplasmic localization by miR-708-5p would promote cell apoptosis, we carried out a co-immunoprecipitation assay to capture the endogenous p21/procaspase-3 complexes in the miR-708-5p expressing A549 and PG cells and their control cells. The assay showed that p21/procaspase-3 binding was markedly weakened in the miR-708-5p expressing A549 and PG cells when compared to their corresponding control cells (Figure 4D, 4E). The co-immunoblotting assay also revealed much higher abundance of active caspase-3 in the miR-708-5p expressing A549 and PG cells than in the control cells (Figure 4D). These observations support the blocked cytoplasmic localization of p21 as the mechanism responsible for miR-708-5p induced apoptosis.

We used immunocytochemistry to survey relationship between cytoplasmic pT145-p21 and the actin cytoskeleton in the A549, PG cells transfected with miR-708-5p mimics and 95C cells transfected with antagomir-708-5p. The miR-708-5p expressing cells showed an extensive network of stress fibers, lower expression of cytoplasmic pT145-p21, and failed to induce the actin rearrangement as observed in the control cells (Figure 4F). In contrast, antagomir-708-5p cells had markedly fewer actin stress fibers, higher expression of cytoplasmic pT145-p21 (Figure 4F). These results suggest that cytoskeleton reorganization in lung cancer cells may be blocked by miR-708-5p. Taken together, these results indicate that miR-708-5p impairs lung cancer cell migration and promotes cancer cell apoptosis by inhibiting the cytoplasmic localization of p21.

### miR-708-5p expression weakens the stem cell like properties of lung cancer cells

**Figure 5:**
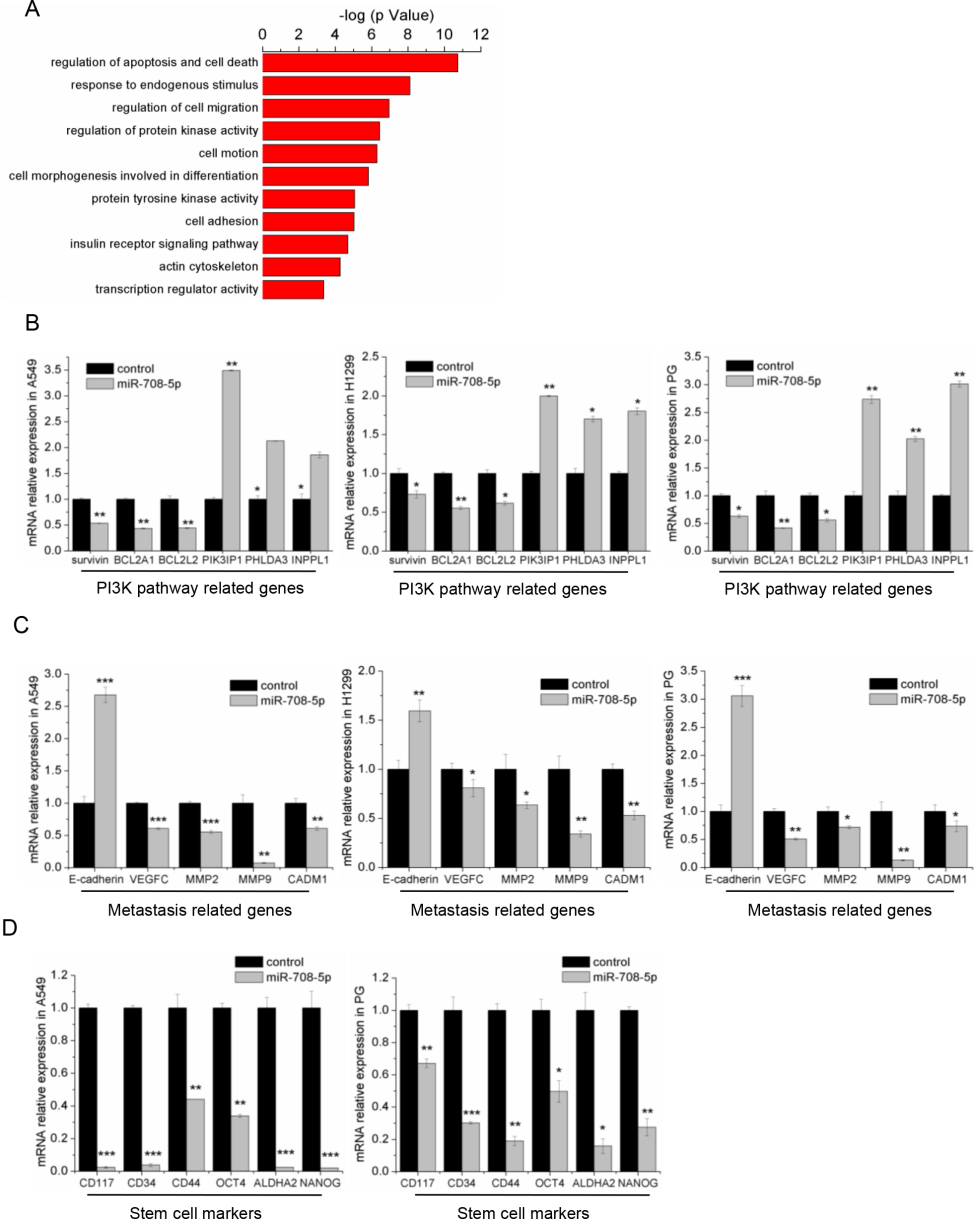
miR-708-5p inhibits the metastasis related pathway and stem cell markers (**A**) The functional annotation clusters in universal genes differentially expressed in the A549 cells treated with miR-708-5p or control mimics. The annotation clusters were predicted from RNA-seq data of the cells by use of the DAVID. (**B**) The differential expression of PI3K pathway related genes identified in the deep sequencing data was confirmed in the miR-708-5p overexpressing A549, H1299, PG cells and their control cells by qRT-PCR. β-actin was used as a normalized control, and *P < 0.05, **P < 0.01. (**C**) The differentially expressed metastasis-related genes identified in the deep sequencing data were confirmed in the miR-708-5p overexpressing A549, H1299, PG cells and their control cells by qRT-PCR. β-actin was used as a normalized control, and *P < 0.05, **P < 0.01, ***P < 0.001. (**D**) The differentially expressed stem cell marker genes identified from the deep sequencing data were confirmed in the miR-708-5p overexpressing A549, PG cells and their control cells by qRT-PCR. β-actin was used as a normalized control, and *P < 0.05, **P < 0.01, ***P < 0.001.

To determine the cellular pathways regulated by miR-708-5p, we identified 599 genes with significant differential expression from RNA-seq data of the miR-708-5p overexpressing A549 cells and control cells. Bioinformatic analysis of global genes using DAVID [29] revealed that miR-708-5p may alter the apoptosis and migration pathways, including EGF receptor, insulin receptor, as well as PI3K/AKT (Figure 5A). Using RNA-seq data collected from the present study, we selected some related genes to further confirm their expression by qRT-PCR. In agreement with our RNA-seq data, the qRT-PCR assay showed that overexpression of miR-708-5p increased expression of the PI3K/AKT pathway repressor genes, PIK3IP1, PHLDA3 and INPPL1, but suppressed expression of the pathway's effector genes, BCL2A1, BCL2L2 and survivin, in the A549, H1299 and PG cells (Figure 5B). Again, the qRT-PCR assay confirmed that the genes (MMP2, MMP9, CADM1 and VEGFC) promoting metastasis were substantially down-regulated in these miR-708-5p expressing cells, while E-cadherin, which inhibits metastasis, was up-regulated (Figure 5C). Interestingly, some cancer stem cell (CSC) associated transcripts, such as CD117, CD34, Oct-4, CD44, ALDHA2 and Nanog, were down regulated in the A549 and PG cells expressing miR-708-5p (Figure 5D). However, no statistically significant difference was detected for these genes in the H1299 cell line (Supplementary Figure S4). These results indicate that miR-708-5p inhibited the lung cancer stem cell-like properties. The CSCs have been proposed to be responsible for cancer progression and metastasis [30], and have great potential for the development of CSC-related therapies [31]. We thus proposed here to suppress the cancer stem cell like properties through overexpressing miR-708-5p in lung cancer cells as a potential new targeted therapy against lung cancers.

### miR-708-5p mediated replacement therapy in a mouse lung tumour model

**Figure 6:**
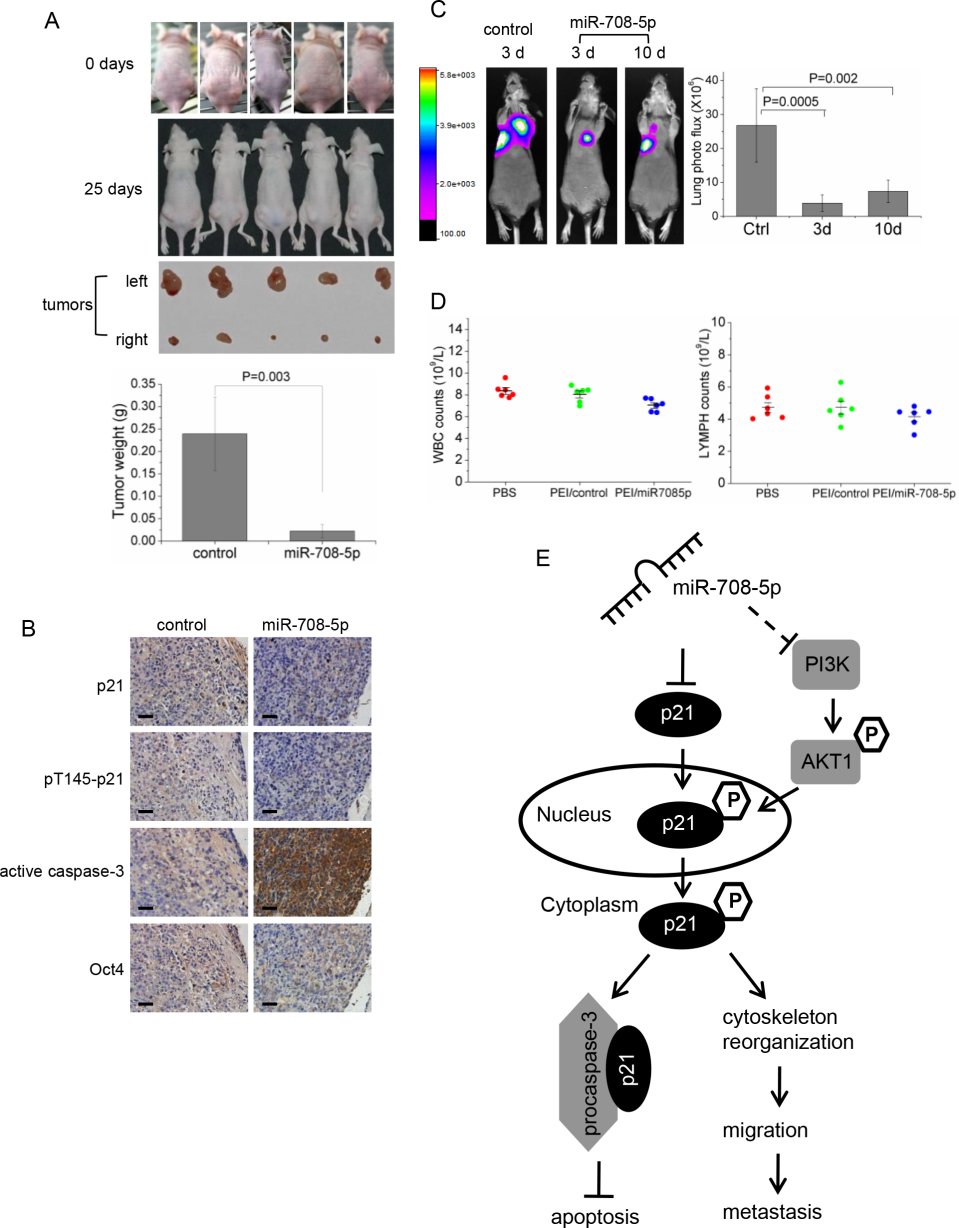
Anti-tumor assays of the replacement therapy for PEI/miR-708-5p treatment in the A549 lung cancer mouse model (**A**) The A549 cells were subcutaneously injected into nude mice to form solid tumors, and then intra-tumorous delivery of the PEI/miR-708-5p was conducted continuously for 3 weeks. Generated subcutaneous tumors in the right and left flank areas, where the right subcutaneous tumors were injected with the PEI/miR-708-5p and the left tumors with PEI/control, were compared with each other. Upper: representative images of mice before intra-tumorous injections were started. Middle: representative images of mice and subcutaneous tumors dissected at day 25 after the PEI/miR-708-5p treatment. Lower: means and s.d. of weight of subcutaneous tumors after intra-tumoral injections (*n* = 5 for each group). (**B**) Immunohistochemical staining of p21, pT145-p21, active caspase-3, Oct-4 in tumor tissues dissected from nude mice treated with the PEI/miR-708-5p or PEI/control. Scale bars, 80 μm. (**C**) Metastasis of the A549 lung cancer cells after the PEI/miR-708-5p replacement treatment. Left: Presented are bioluminescence images of animals showing lung metastasis at day 25 after PEI/miR-708-5p replacement treatment. The treatments were intravenously delivered twice weekly for 3 weeks, starting at 3 day or 10 day after tumor cell implantation. The color scale bar depicts the photon flux (photons per second) emitted from these mice. Right: Quantitation of lung metastases assessed by bioluminescences measurements (*n* = 6 per group). Data represent mean ± s.d. (**D**) Toxicity assessment of intravenous delivery of PEI/miR-708-5p, PEI/control or PBS in normal mice. White blood cell (WBC) and lymphocyte (LYMPH) count. Data were presented as mean and s.d. (*n* = 6 per group). (**E**) A proposed model for the miR-708-5p-mediated pathway regulating NSCLC metastasis.

Based on our *in vitro* and *in vivo* assays that confirmed the anti-metastatic and anti-cancer stem cell activities of miR-708-5p in NSCLC, we tested whether synthetic miR-708-5p mimics could have potential for replacement therapy in a mouse lung cancer model. We firstly explored the antitumor effect of the synthetic miR-708-5p mimic in the lung cancer xenograft model. Nude mice were subcutaneously inoculated with the same volume of A549 cells in the right and left flank areas, and then intra-tumorally administered with miRNA or control mimics (fully described in Methods). All mice were sacrificed after 25 days following inoculation. As shown in Figure 6A and Supplementary Figure S5A, mice injected with the PEI/control showed rapid tumour growth, with an approximately 20-fold increase in tumour volume over 25 days when compared to mice injected with PEI/miR-708-5p. We compared expression of the miRNA between the treated group and the control group, and observed that the miRNA expressed approximately 5000-fold higher in the miR-708-5p mimic than in the control tumours (Supplementary Figure S5B). Additionally, immunohistochemical analysis of these tumours revealed significantly increased active caspase-3 and decreased expression of p21, pT145-p21, and Oct-4 (CSC associated genes) in the tumours injected with miR-708-5p (Figure 6B). These observations strongly support the marked anti-tumor and anti-cancer stem cell effects of the miR-708-5p treatment.

To evaluate the anti-metastatic effects of the miR-708-5p mimic treatment, we implanted the A549 cells with a luciferase reporter into nude mice through tail vein injection, as described in Methods. We began the miR-708-5p replacement treatment at day 3 or day 10 after cancer cell implantation. After a 25-day course of delivery treatment, *in vivo* bioluminescence imaging showed that administration of PEI/miR-708-5p into mice attenuated lung metastases when the treatment was performed at day 3 or day 10 (Figure 6C). To focus on the systemic PEI/miR-708-5p delivery, we compared miR-708-5p expression in the livers and lungs of animals injected with either PEI/miR-708-5p or the PEI/control. The expression levels of miR-708-5p in livers and lungs of the PEI/miR-708-5p group were approximately 30 and 120 fold higher than that in the control group, respectively (Supplementary Figure S5C, S5D). Thus, systemic delivery of PEI/miR-708-5p complexes appeared to be a potent approach to suppress metastasis of mouse lung cancer cells.

To assess the potential toxicity of the PEI/miR-708-5p treatment, we exposed healthy mice upon the complex using the same dosing regimen as described in the above therapy study. Intravenous delivery of PEI/miR-708-5p increased miR-708-5p levels in liver tissues, whereas the PEI/control did not modulate miR-708-5p levels relative to PBS (Supplementary Figure S6A). All three groups of mice tolerated the procedure well and exhibited normal behaviours. Body weights were not affected by the PEI/miR-708-5p treatment (Supplementary Figure S6B). Histo-pathological examination of the livers revealed no steatosis, portal or lobular inflammation, necrosis, fibrosis, nor biliary change in any of the three groups (Supplementary Figure S6C). White blood cells (WBC) and lymphocytes (LYMPH) in the PEI/miR-708-5p group of mice showed a slight decrease, but remained in the normal range when compared to both the PBS group and PEI/control group (Figure 6D). Also, a cell cycle and proliferation assay showed that miR-708-5p has no effect on the growth of normal human lung cell WI-38 *in vitro* (Supplementary Figure S6D, S6E). Thus, the PEI/miR-708-5p treatment involves no apparent toxic effects on the treated animals and normal human cells.

## DISCUSSION

This article provides the first report of miR-708-5p as an anti-metastatic miRNA and a direct negative regulator of the cyclin-dependent kinase inhibitor, p21, in human non-small cell lung cancers. It demonstrates that miR-708-5p can suppress not only total p21, but also the cytoplasmic localization of p21, which in turn elevates apoptosis and weakens actin rearrangement, leading to decreased cell motility (Figure 6E). Moreover, miR-708-5p suppresses both the stem cell like properties of lung cancer cells and the PI3K/AKT pathway. For its dual roles in anti-metastasis and conferring anti-stem cell properties, miR-708-5p is proposed here to be a suitable candidate for miRNA replacement therapy. The *in vivo* miR-708-5p restoration in the lung tumor xenografts by intra-tumorous delivery has been found to restore normalcy and inhibit tumor formation in the lung cancer mouse model. Likewise, restoration of miR-708-5p does also inhibit metastasis in the mouse lung metastasis model.

Despite significant advances in cancer therapy over the past 50 years, developing treatments for metastatic solid cancers remains one of the greatest challenges in modern clinical cancer research. Survival of patients with such malignancies is typically limited to months only. In the era of targeted therapy, a prominent and well-defined role of the PI3K/AKT pathway in modulating cancer cell growth and survival has motivated development of PI3K/AKT pathway inhibitors as a promising and effective therapy for malignant solid cancers [27, 32]. This therapeutic strategy has been developed into several successful clinical and/or pharmaceutical applications [33–35]. But the limitation of these inhibitors lies in the development of drug resistance for the signaling feedback loop in cancer cells. Our RNA-seq data shows that miR-708-5p impairs the PI3K/AKT pathway through down-regulating multiple receptor tyrosine kinases, such as the EGF receptor family genes and insulin receptor family genes, revealing that miR-708-5p can inactivate the multiple feedback loop of the PI3K/AKT pathway and may be substantially more effective than single-agent PI3K pathway inhibitors. miR-708-5p mediated replacement therapy may therefore represent a promising compensator or even an alternative to the current PI3K pathway inhibitors. In addition to inhibiting the PI3K pathway, miR-708-5p suppresses several other intracellular pathways, in turn inhibiting cancer stem cells, cancer cell migration and survival. These findings indicate that miR-708-5p may enable multiple antitumor effects by specifically interfering with several oncogenic pathways, thereby preventing tumor cells from escaping drug targeting. Furthermore, the cytoplasmic expression of p21 enables cancer cells to escape drug-induced apoptosis and can result in drug resistance during chemotherapeutic treatment [36–39]. Thus, if chemotherapeutic treatment of cancer cells could be integrated with use of a miR-708-5p mimic, the tumors may then become more susceptible to the drug targeting and apoptosis, improving the efficiency of chemotherapy.

PEI is the synthetic polymers, which are able to form non-covalent complexes with DNAs or RNAs. These nanoscale complexes enable protection of DNAs or RNAs from nucleolytic degradation, their efficient cellular uptake through endocytosis and intracellular release through the proton sponge effect [40]. Being nontoxic, linear PEI, one kind of PEI polymers, is under clinical trials [41]. This paper presents the first linear PEI-based miRNA replacement therapy for lung cancer in a mouse model. Our data of systemic delivery of the PEI/miR-708-5p complexes demonstrate that miR-708-5p expression in mouse lung cells was 4 to 6 folds higher than that in the liver cells, suggesting that PEI/miRNA is an efficient delivery platform for treatment of lung diseases with little or no systemic side effects. Also, the miR-708-5p replacement therapy experiment demonstrates that the PEI-dependent delivery of miRNAs into tumours represents an efficient therapeutic schedule for lung cancer. Despite the tested high efficacy of the miR-708-5p mediated gene therapy, further knowledge about the PEI-based delivery systems may need to be accumulated before clinical trials will be initiated with humans.

## MATERIALS AND METHODS

### Cell lines and tissue samples

Cell lines H520, H2170, H226, H1703, A549, H1299, and WI-38 were obtained from American Type Culture Collection and cultured under conditions provided by the manufacturer. Cell lines PG, 95C and QG56 were obtained from the Cell Bank of Type Culture Collection of the Chinese Academy of Science (CBTCCCAS, Shanghai). All the cells were authenticated by short tandem repeat DNA profiling upon initial receipt and periodically thereafter, and were propagated for less than 6 months after resuscitation. These cells were cultured at 37°C under 5% CO_2_ in RPMI-1640 (Invitrogen) supplemented with 10% FBS (Thermo scientific), and penicillin/streptomycin (Thermo scientific).

72 NSCLC samples including 36 Adc and 36 Sqc, were collected between 2003 and 2008 at Shanghai pulmonary hospital in China. The clinicopathologic characteristics of patients were listed in Supplementary Table S1. Another 66 frozen human primary NSCLC tissues (28 Adc and 38 Sqc) were obtained at Xijing Hospital in 2013. Tumors were classified according to the World Health Organization pathologic classification system. All patients provided informed consent and did not receive any therapy before surgery.

### Antibodies

Antibodies used in the present study are as following: p21 (Abcam), pT145-p21 (Abcam), AKT1 (Santa Cruz), pS473-AKT1 (Santa Cruz), Vimentin (Abcam), β-catenin (Abcam), GAPDH (Abcam), β-actin (Sigma), cleaved PARP (Abcam), active caspase-3 (Cell signaling), procaspase-3 (Abcam), Oct4 (Abcam), E-cadherin (R & D), S1PR1 (Abcam).

### Isolation of RNA, reverse transcription and real-time PCR quantification

Total RNA was extracted from frozen tissues or cultured cells using Trizol total RNA isolation reagent (Invitrogen), as the manufacturer's instructions. cDNA was synthesized from total RNA or purified small RNAs using gene-specific primers or random hexamers with the SUPERSCRIPT III Reverse Transcriptase Kit (Invitrogen), according to the manufacturer's instructions. Real-time PCR was performed using an Applied Biosystems 7500. The relative expression level was calculated from a relative standard curve obtained by using log dilutions of cDNA containing the mRNA or miRNA of interest. The average of two independent analyses for each gene and sample was calculated and normalized to the endogenous reference control gene ACTIN or U6 small nuclear RNA.

### miRNA/siRNA transfection

Cells were planted in growth medium without antibiotics approximately 24 hours before transfections. Transient transfections of miRNA mimics/antagomir/siRNA were carried out by using lipofectamine 2000 (Invitrogen) according to the manufacturer's protocol. All miRNA mimic/antagormir/siRNA transfections were for 48 hours.

### Lentivirus mediated cell transfection and transduction

The miR-708-5p sponge was constructed with a method modified from previous reports [42]. To establish immortal cell lines with miR-708-5p sponge or control sponge, we employed a lentivirus-mediated cell transformation technique. Recombinant lentiviruses were produced by co-transfecting human embryonic kidney (HEK) 293T cells with the miR-708-5p-sponge lentiviral expression plasmid and packaging plasmids (delta 8.9 and VSVG) using Lipofectamine 2000 (Invitrogen) as a transfection reagent. HEK 293T cells were cultured in DMEM supplemented with 10% FCS in a 37°C incubator with 5% CO_2_. Infectious lentiviruses were collected 48 h after transfection. The supernatant was centrifuged to remove cell debris and filtered through 0.45 μm filters (Millipore). Cells were infected with the collected lentivirus.

### Luciferase assay

For target gene assays, the 3′UTR of human p21 was amplified using PCR and cloned into a pGL3 vector. Cells of 70% confluence in 24-well plates were transfected using Lipofectamine 2000. The constructed pGL3 vector (100 ng), 5 ng of pRL-SV40 Renilla luciferase construct (for normalization), and 100 ng of control mimics or miR-708-5p mimics were cotransfected per well. Cell extracts were prepared 36 h after transfection, and the luciferase activity was measured using the Dual luciferase reporter assay system (Promega).

### Wound healing assay

Cells were seeded in 24-well plates and cultured until confluent. Cells were then serum-starved for overnight before wounding. The cell layers were scraped with a plastic pipette tip and washed three times with serum-free medium. The remaining cell was incubated 24 h or 48 h to allow cells to migrate into the cleared space. To quantify cell migration, phase-contrast images of identical location in each wound were taken at 0 h, 24 h and 48 h after wounding. The distance of cell migration was measured.

### MTT assay for cell proliferation

Cells were grown in 96-well plants in volumes of 200 μL of medium per well. MTT (5 mg/ml, Sigma) was added to each well in a volume of 20 μL. After a further period of incubation for 4 h at 37°C, the medium was aspirated from the wells as completely as possible without disturbing the formazan crystals, and 200 μL DMSO was added to each well. The plates were shaken for 10 min to dissolve the purple formazan crystals. Then the optical density was recorded using a micro-plate reader (Bio-Tek Instruments) at 570 nm.

### Cell cycle analysis stained by PI

Cells were suspended in 400 μL PI (Propidium Iodide)/Triton X-100 staining solution and incubated 37°C for 15 minutes, then detected by flow cytometer (BD Biosciences). PI/Triton X-100 staining solution: 0.1% (V/V) Triton X-100 (Sigma) in PBS add DNase-free RNase A (Sigma) and 500 μg/ml PI (Sigma).

### Migration and invasion assays

*In vitro* cell migration assays were performed as described previously using Trans-well chambers (8 μM pore size; Costar). Cells were allowed to grow to subconfluency (∼75–80%) and were serum-starved for 24 h. After detachment with trypsin, cells were washed with PBS and resuspended in serum-free medium. Next, 100 μl cell suspension (2 × 10^6^ cells/mL) was added to the upper chamber. Complete medium was added to the bottom wells of the chambers. For the screen, the cells that had not migrated after 24 h were removed from the upper face of the filters using cotton swabs, but the cells that had migrated were fixed with 5% glutaraldehyde solution to determine the number of migratory cells. The lower surfaces of the filters were stained with 0.25% Trypan Blue. Images of six different × 10 fields were captured from each membrane and the number of migratory cells was counted. The mean of triplicate assays for each experimental condition was used. Similar inserts coated with Matrigel were used to evaluate cell invasive potential in the invasion assay.

### Immunoblotting

Total protein was separated by 10∼15% sodium dodecyl sulfate-polyacrylamide gel electrophoresis and transferred to PVDF membranes. After blocking in 5% nonfat milk, the membrane was incubated with the primary antibodies, prior to washing and exposure to peroxidase-conjugated secondary antibodies. Ultimately, the membrane was treated with chemiluminescence reagents (Santa Cruz) as per the manufacturer's instructions, and the image was developed on X-ray films.

### Apoptosis assays

FACS analysis for apoptosis was done 72 hours after transfection, using Annexin V-FLOUS/PI Kit (Roche), according to the manufacturer's protocol. Stained cells were immediately analyzed with a flow cytometer (BD Biosciences).

### Actin stress fiber staining and immunocytochemistry

Cells were grown on glass coverslips for 24 h and serum starved for 48 h, then stimulated by exposure to 10% serum for 6 h. Cells were fixed in 4% paraformaldehyde for 20 min and permeabilized with 0.1% Triton X-100 for 15 min at room temperature. The coverslips were incubated in the dark with pT145-p21 antibody (Abcam) at 4°C overnight, then counterstaining with FITC goat secondary antibodies. Next, the coverslips were incubated with 100 nM rhodamine phalloidin (Cytoskeleton) at room temperature for 30 min. Nuclei were counterstained with 100 nM DAPI. The coverslips were rinsed in PBS and inverted on a drop of anti-fade mounting media on a glass slide. Then these slides were sealed with nail polish and viewed under the confocal microscope.

### Immunohistochemistry

Paraffin sections were deparaffinized. Immunostaining using monoclonal antibodies p21, pT145-p21, active caspase-3 and Oct-4 were performed according to the methods provided in the immunoCruz Staining System Kit (Santa Cruz Biotechnology). Briefly, hydrated tissue sections were steamed for 30 min in 0.1% citrate acid and blocked in serum for 20 min at room temperature. Samples were subsequently incubated with the indicated antibody for 1 h and with biotinylated secondary antibody for 30 min, and then visualized using chromogen 3′3′-diaminobenzidine (DAB). Slides were counterstained with Mayer's hematoxylin for 20 sec and rinsed abundantly in H_2_O before dehydration and mounting.

### Coimmunoprecipitations

A549 and PG cells synchronized by serum deprivation for 48 h were restimulated by exposure to 10% serum for 6 h. Cells were lysed in 500 μL lysis buffer (cell signaling) for a 100 mm plate. For each IP, 1 μg of the appropriate antibody, 2 mg lysate proteins, and 20 μL of protein A sepharose beads (Amersham) were incubated for 3 h at 4°C. Immunoprecipitates were rinsed three times in lysis buffer, and 10 μL of 4 × sample buffer was added to the bead pellet. Immunoprecipitates, as well as 10 μL of the corresponding lysates for protein loading, were subjected to immunoblotting as described above.

### RNA-seq library construction, sequencing and data analysis

Total RNA was evaluated the integrity and concentration using Agilent Technologies 2100 bioanalyzer. For each sample, 2 ug of total RNA with a RIN value more than 8.0, was imputed into the procedure of RNA-seq library preparation as per Illumina TruSeq RNA Sample Preparation protocol. RNA-seq libraries were sequenced using the Illumina HiSeq 2000 instrument as per manufacturer's instructions. Sequencing of libraries was performed up to 2 × 101 cycles. Image analysis and base calling were performed with the standard Illumina pipeline version RTA 2.8.0.

The sequencing reads containing either N over 3% or low quality bases (< 18) over 50% of the full length in each end were removed. All the clean reads were then aligned to the UCSC (the University of California Santa) *Homo sapiens* reference genome using TopHat v1.3.3 with default parameters [43]. The expression levels of the transcripts were quantified by Mapped Fragments per Kilobase of Exon model per Million mapped reads (FPKM) using Cufflinks [44]. Differential expression gene was identified with the output q ≥ 0.8 using non-parameter test provided by NOISeq [45]. The panel of differential expression genes was performed functional clustering and enrichment analyses using the Database for Annotation, Visualization and Integrated Discovery (DAVID) [29].

### Mouse experiments

All animal experiments were performed according to the protocol of the Fudan Committee on Animal Care using six-week-old to eight-week-old female BALB/c nude mice. 6×10^6^ QG56 cells transduced with miR-708-5p sponge or control sponge were injected into the caudal vein. Lung metastatic colonization was monitored and quantified after 8 weeks. Each group was comprised of 6 mice unless otherwise stated.

To examine the antitumor effects of miR-708-5p, nude mice (*n* = 5) received subcutaneous injections of 3 × 10^6^ A549 cells in the right and left flank areas in a volume of 100 μL. When tumors reached an average volume of 50 to 100 mm^3^, 10 μg of synthetic miRNA (miR-708-5p or control) mimics (Genephama, China) mixed with 1.2 μL of *in vivo* jet polyethylenimine (PEI) transfection reagent (Polyplus Transfection, France) was delivered intra-tumorously twice a week for 3 weeks. Mice were sacrificed after the last treatment, and the tumors generated were used in further analysis. PEI is one of the promising polycations because it condenses RNA or DNA and the resulting PEI/RNA (PEI/DNA) complex can act as proton sponge, thus enabling RNA or DNA delivery into cytoplasm via endosomes.

To assess the anti-metastatic effects of miR-708-5p, nude mice were implanted with A549 cells (6 × 10^6^ cells per mouse). The miRNA/PEI treatment started 3 days or 10 days after tumor cell implantation: miR-708-5p or control mimics (40 μg) mixed with 4.8 μL of *in vivo* jet PEI were injected via the tail vein, twice weekly for 3 weeks. Pulmonary metastases were monitored by live animal BLI (Xenogen IVIS system) 25 days after tumor cell implantation.

### Statistical analysis

All values were presented as mean ± standard deviation (SD), and Student's *t*-test (two-tailed) was used to compare two independent groups. The Bartlett test and/or Mann-Whitney-Wilcoxon test was implemented to evaluate differential expression of miR-708-5p between metastatic and non-metastatic lung cancer samples. Differences in patient characteristics between different miR-708-5p groups were tested with Kruskal-Wallis test. Expression data was normalized to the endogenous reference U6 before statistical analysis. Survival curves were plotted using the Kaplan-Meier method, and survival difference was assessed by the log-rank test using the median of each individual miRNA as a cutoff. *P* value < 0.05 was considered statistically.

## SUPPLEMENTARY MATERIALS FIGURES AND TABLE


