# Supplementary material for: MicroRNA-708-5p acts as a therapeutic agent against metastatic lung cancer
Source: Oncotarget. 2015 Dec 14;7(3):2417–32. doi: 10.18632/oncotarget.6594 (PMC4823045; doi:10.18632/oncotarget.6594)
Supplement: Supplementary file 1 [file oncotarget-07-2417-s001.pdf]

MicroRNA-708-5p acts as a therapeutic agent against metastatic lung cancer

Supplementary Materials

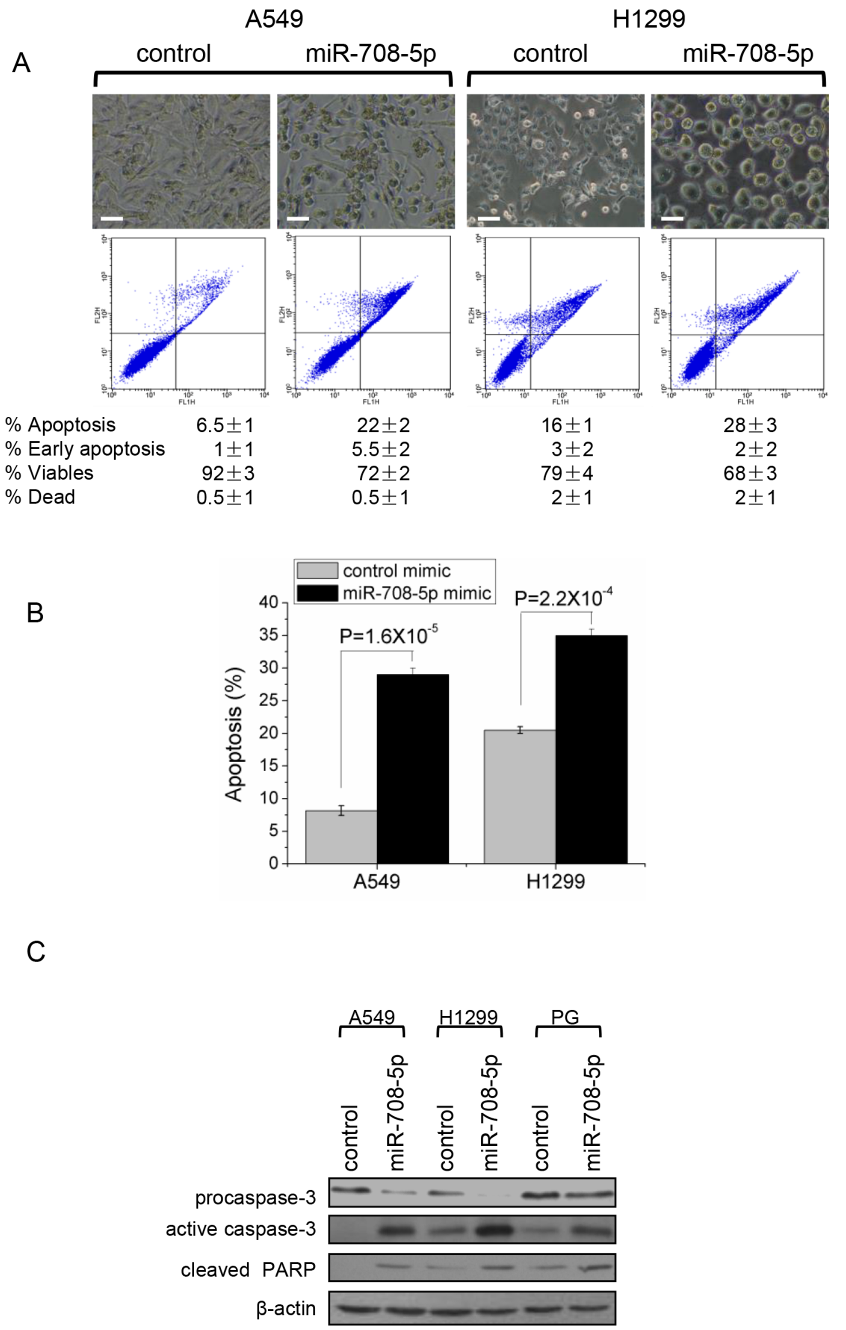

Supplementary Figure S1: miR-708-5p induced cell apoptosis in lung cancer cells. (**A**) Apoptosis assay was performed in the A549 and H1299 cells transfected with either control mimics or miR-708-5p mimics after serum deprivation for 48 h. Upper group: altered morphology of the A549 and H1299 cells transfected with the control or miR-708-5p mimics. The assays were assessed by phase-contrast microscopy. Scale bars, 80 µm. Lower group: apoptosis assays of the A549 and H1299 cells using the Annexin V/PI kit after the control or miR-708-5p mimics treatments and the assays were detected by flow cytometry. (**B**) Proportions of average apoptosis (including both early and late apoptosis) in the comparing groups of A549 and H1299 cells. (**C**) Immunoblotting analysis for procaspase-3, active caspase-3 and cleaved PARP in the control or miR-708-5p-transfected A549, H1299 and PG cells. β-actin was used as a loading control.

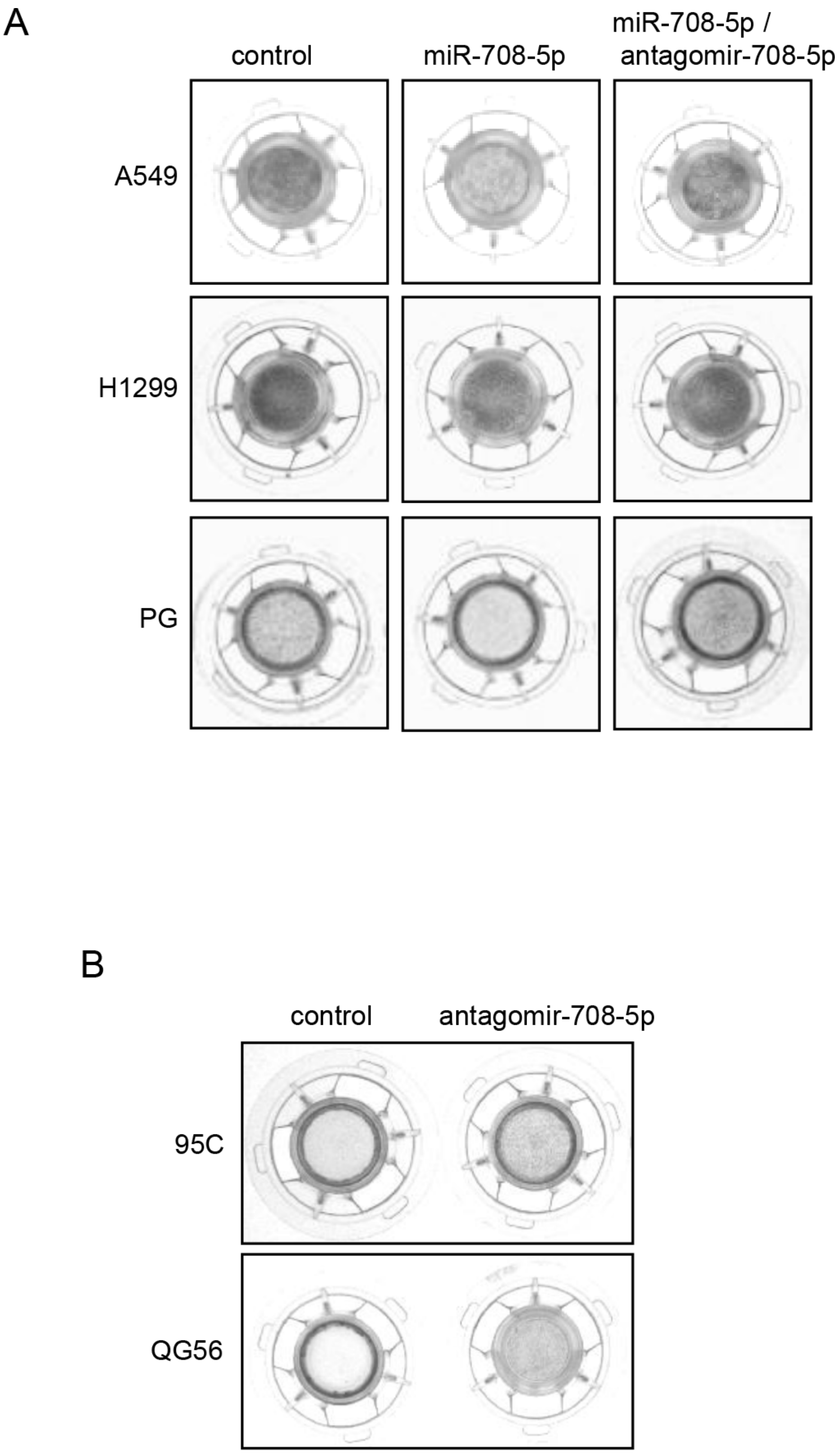

Supplementary Figure S2: Overexpression of miR-708-5p suppressed cells migration and this effect was abolished in the present of antagomir-708-5p. (**A**) Transwell migration assays of A549, H1299 and PG cell were performed after transfection by control mimics, miR-708-5p mimics, or miR-708-5p/antagomir-708-5p. (**B**) Inhibition of miR-708-5p promotes cell migration. Transwell migration assays of 95C and QG56 cells were performed after transfection by control antagomir or antagomir-708-5p.

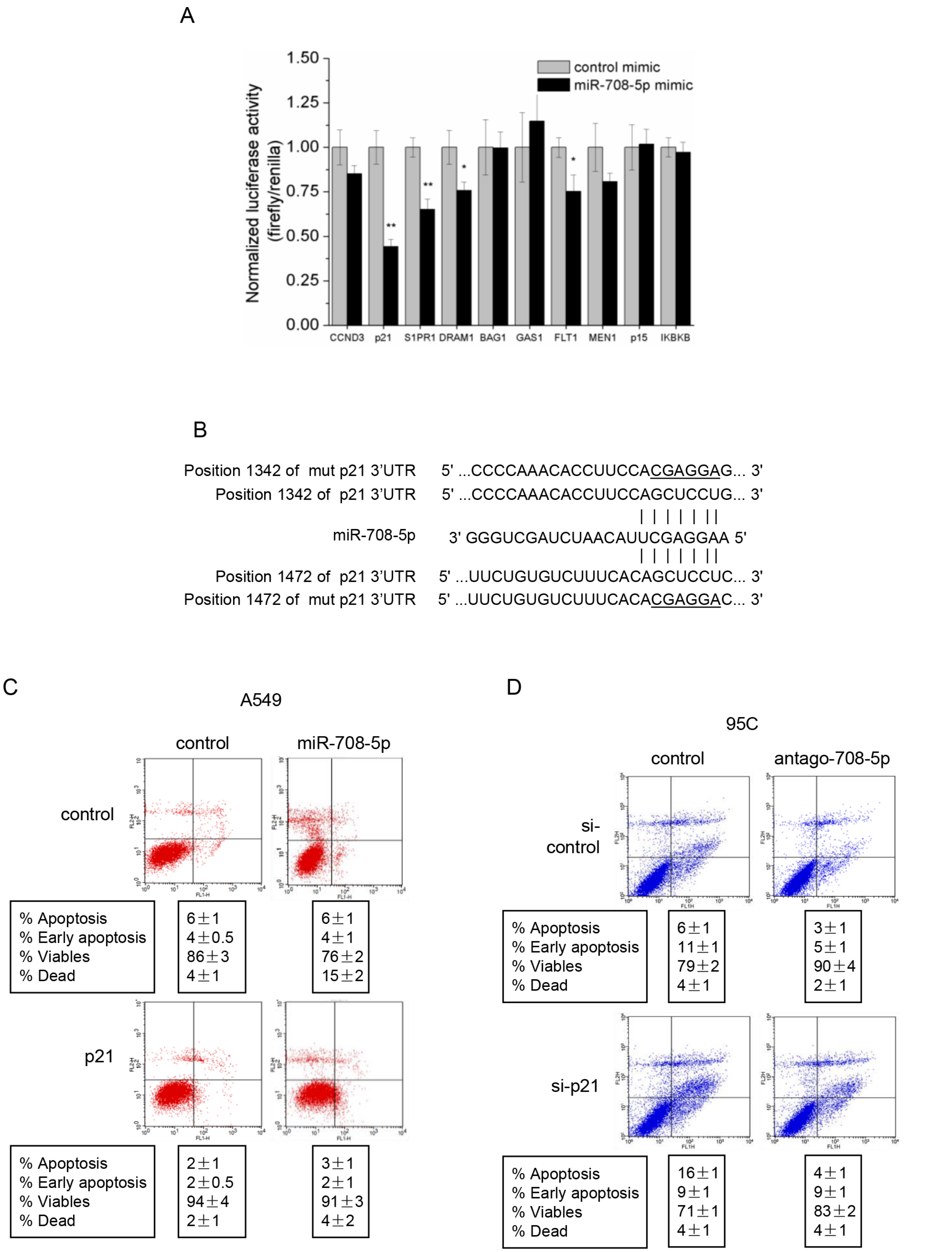

Supplementary Figure S3: Target genes analysis for miR-708-5p. (**A**) Luciferase activity assay with respective luciferase constructs of 10 genes' wild–type 3'UTR which were co-transfected with the miR-708-5p or control mimics, where *stands for significance at $P < 0.05$, and **at $P < 0.01$. (**B**) Predicted duplex formation between human p21 3'UTR and miR-708-5p. (**C**, **D**) Rescued apoptosis assay in A549 (C) and 95C (D). Apoptosis assays using the Annexin V/PI kit were detected by flow cytometry. A549 was overexpressed p21 in the presence of miR-708-5p and 95C was silenced p21 by siRNA in the downregulation of miR-708-5p.

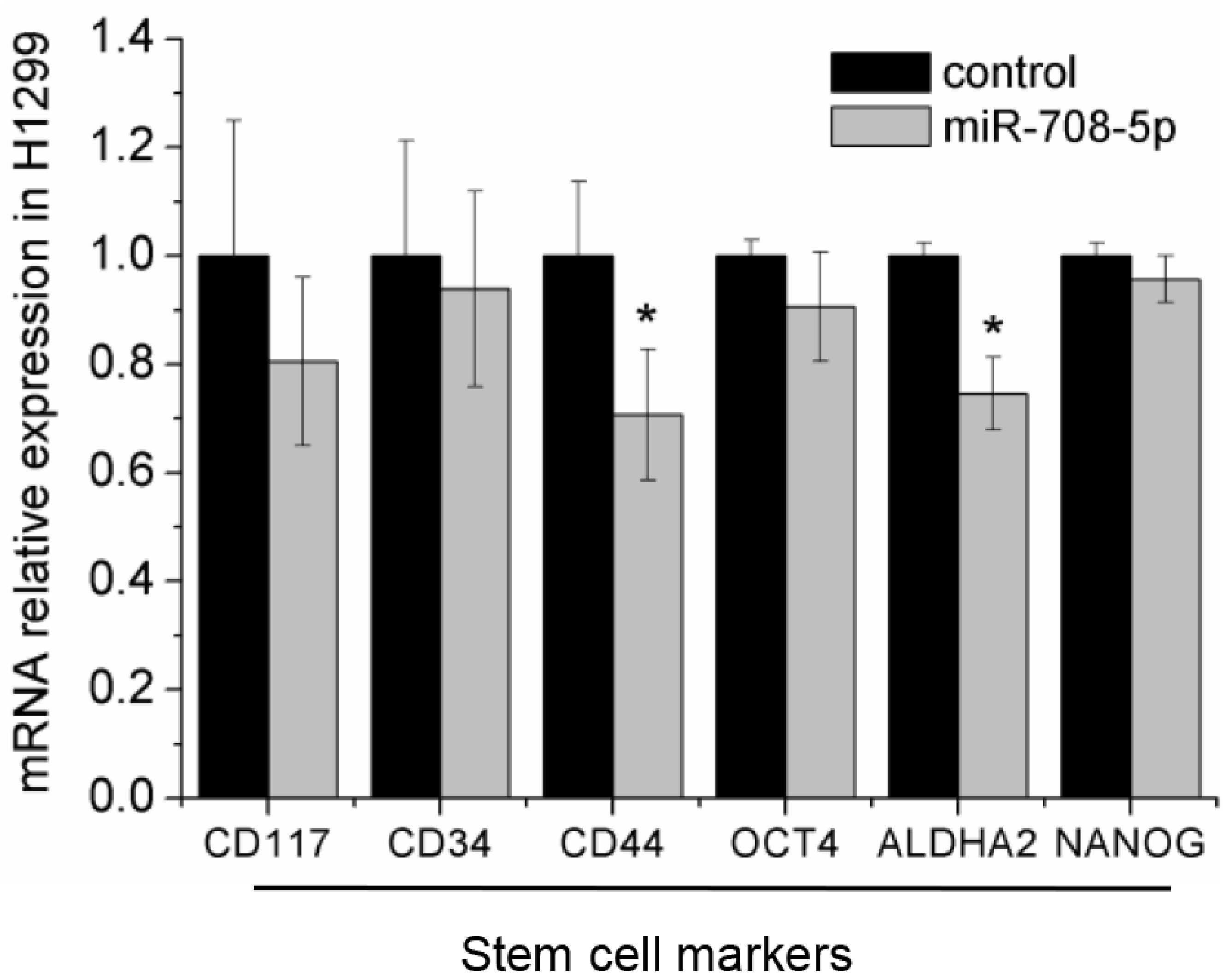

Supplementary Figure S4: The differential expression stem cell marker genes identified in the deep sequencing was confirmed in miR-708-5p overexpressing H1299 cells and their control cells by qRT-PCR. β-actin was used as a normalized control. *$P < 0.05$.

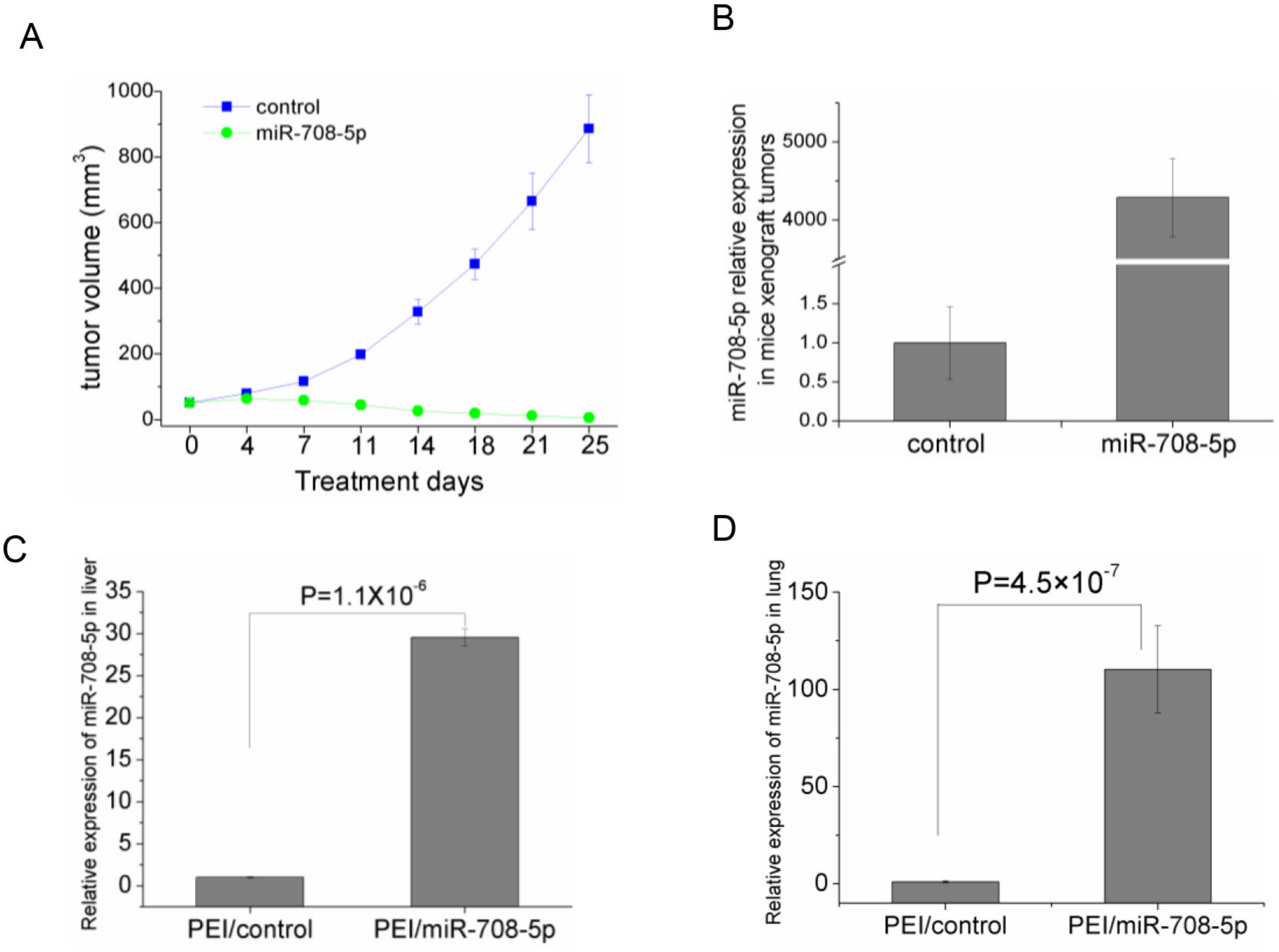

Supplementary Figure S5: Some assays of replacement therapy for PEI/miR-708-5p treatment in the A549 lung cancer mouse model. (**A**) Tumor volumes following PEI/miR-708-5p administration in comparison with the PEI/control treated mice. (**B**) Relative expression of miR-708-5p in lung cancer xenografts as assessed by qRT-PCR. U6 was used as a normalized control. (**C**, **D**) qRT-PCR assay of miR-708-5p in livers (C) and lungs (D) of mice undergone tail vein injection for systematically delivering the PEI/miR-708-5p or PEI/control and subsequently treated with the PEI/miR-708-5p or PEI/control. Data were presented as means and s.d. of miR-708-5p expression (n = 6 per group).

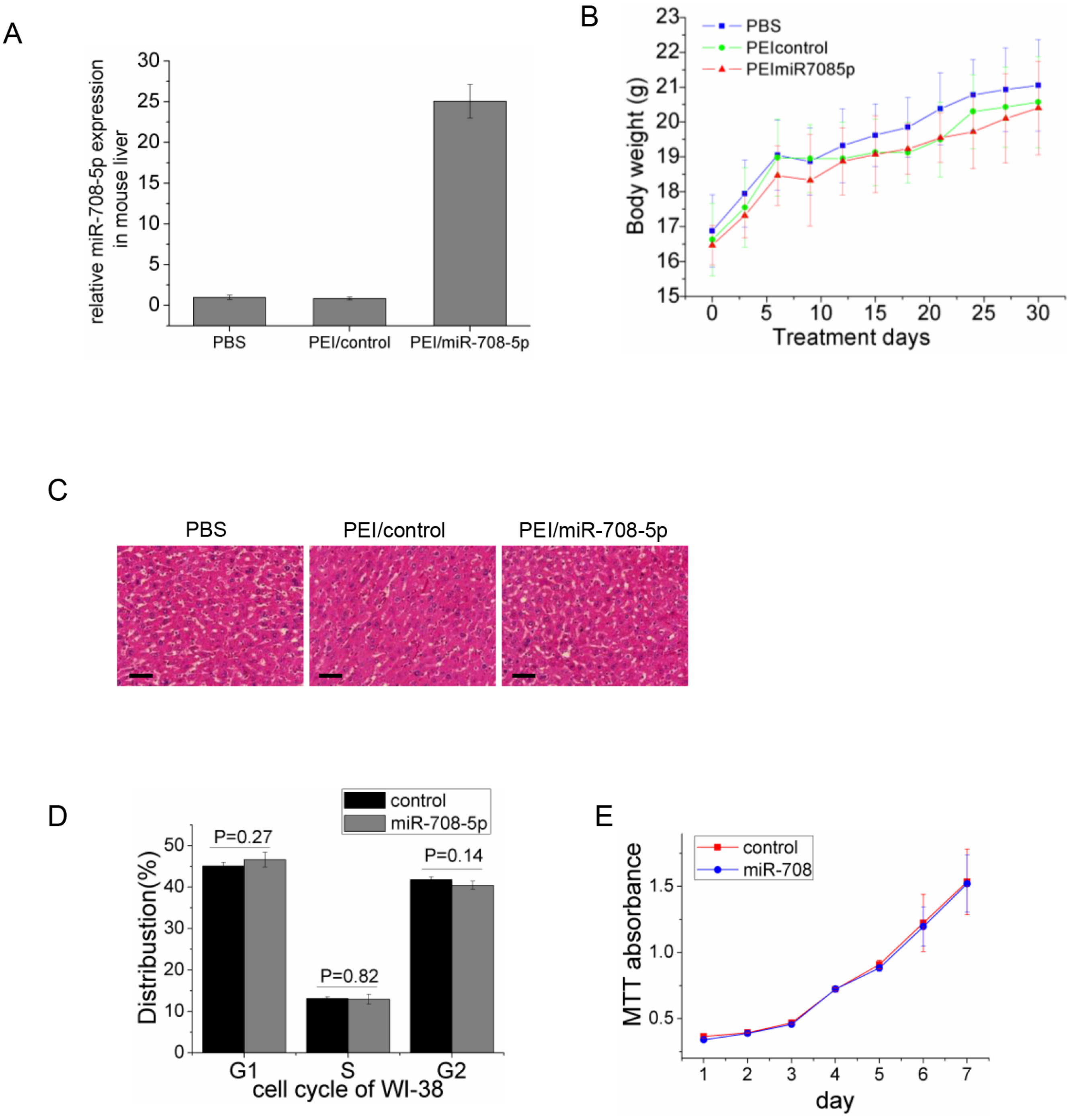

Supplementary Figure S6: Toxicity assessment of intravenous delivery of PEI/miR-708-5p, PEI/control or PBS in normal mice. (**A**) qRT-PCR of miR-708-5p in livers of mice with tail vein injection to deliver systematically PEI/miR-708-5p, PEI/control or PBS (Phosphate buffered saline). (**B**) Total body weight of the tested animals was measured twice a week during the study. (**C**) H & E-stained sections of liver samples. Data were presented as means and s.d. of miR-708-5p expression ($n = 6$ mice per group). Scale bars, 80 µm. (**D**) Cell cycle phase fractions of WI-38 transfected with miR-708-5p mimics and control mimics, which were detected by flow cytometry. (**E**) Cell growth curves of WI-38 transfected with miR-708-5p mimics and control mimics.

Supplementary Table S1: Correlation of miR-708-5p expression with clinicopathological status in 72 cases of patients with NSCLC (Kruskal-Wallis Test)

Characteristics	All No.	Adenocarcinoma	Squamous-cell carcinoma	Hsa-miR-708-5p*	
	n = 72	*n* = 36	*n* = 36	Mean ± SD	*P* value
**Age, years**					0.21145
< 40	4	3	1	1.14722 ± 0.7727	
40–49	5	4	1	1.76337 ± 0.65777	
50–59	28	11	17	1.61311 ± 1.32109	
60–69	21	10	11	2.11985 ± 2.00808	
≥ 70	14	8	6	1.15563 ± 2.00828	
mean ± SD	60.0 ± 9.9	59.6 ± 11.3	60.5 ± 8.2		
median (max-min)	51 (84 − 33)	46 (79 − 33)	47 (84 − 37)		
**Sex**					0.51737
Male	58	22	36	1.6816 ± 1.65242	
Female	14	14	0	1.55256 ± 1.68853	
**Lymph nodes**					0.00124
Negative	32	16	16	2.38423 ± 2.03392	
Positive	40	20	20	1.07433 ± 0.9357	
**TNM stage**					0.00602
I	18	7	11	2.53567 ± 2.3385	
II	31	14	17	1.71704 ± 1.36757	
III	16	10	6	1.09675 ± 0.94156	
IV	7	6	1	0.46072 ± 0.29616	

*We analyzed the association between miR-708-5p expression and the clinicopathological status of NSCLC patients using Kruskal-Wallis Test. The NSCLC patients are 72 cases, including 36 adenocarcinoma and 36 squamous-cell carcinoma.
